# A Critical Reappraisal of Neutrophil Extracellular Traps and NETosis Mimics Based on Differential Requirements for Protein Citrullination

**DOI:** 10.3389/fimmu.2016.00461

**Published:** 2016-11-04

**Authors:** Maximilian F. Konig, Felipe Andrade

**Affiliations:** ^1^Division of Rheumatology, Johns Hopkins University School of Medicine, Baltimore, MD, USA

**Keywords:** NETosis, leukotoxic hypercitrullination, citrullination, rheumatoid arthritis, systemic lupus erythematosus, NADPH oxidase, peptidylarginine deiminase, mitophagy

## Abstract

NETosis, an antimicrobial form of neutrophil cell death, is considered a primary source of citrullinated autoantigens in rheumatoid arthritis (RA) and immunogenic DNA in systemic lupus erythematosus (SLE). Activation of the citrullinating enzyme peptidylarginine deiminase type 4 (PAD4) is believed to be essential for neutrophil extracellular trap (NET) formation and NETosis. PAD4 is therefore viewed as a promising therapeutic target to inhibit the formation of NETs in both diseases. In this review, we examine the evidence for PAD4 activation during NETosis and provide experimental data to suggest that protein citrullination is not a universal feature of NETs. We delineate two distinct biological processes, leukotoxic hypercitrullination (LTH) and defective mitophagy, which have been erroneously classified as “NETosis.” While these NETosis mimics share morphological similarities with NETosis (i.e., extracellular DNA release), they are biologically distinct. As such, these processes can be readily classified by their stimuli, activation of distinct biochemical pathways, the presence of hypercitrullination, and antimicrobial effector function. NETosis is an antimicrobial form of cell death that is NADPH oxidase-dependent and not associated with hypercitrullination. In contrast, LTH is NADPH oxidase-independent and not bactericidal. Rather, LTH represents a bacterial strategy to achieve immune evasion. It is triggered by pore-forming pathways and equivalent signals that cumulate in calcium-dependent hyperactivation of PADs, protein hypercitrullination, and neutrophil death. The generation of citrullinated autoantigens in RA is likely driven by LTH, but not NETosis. Mitochondrial DNA (mtDNA) expulsion, the result of a constitutive defect in mitophagy, represents a second NETosis mimic. In the presence of interferon-α and immune complexes, this process can generate highly interferogenic oxidized mtDNA, which has previously been mistaken for NETosis in SLE. Distinguishing NETosis from LTH and defective mitophagy is paramount to understanding the role of neutrophil damage in immunity and the pathogenesis of human diseases. This provides a framework to design specific inhibitors of these distinct biological processes in human disease.

## Introduction

### The History of NETs: Discovery and Misconceptions

Following Elias Metchnikoff’s initial proposal that wandering cells (“Wanderzellen”), known today as neutrophils, phagocytose and kill bacteria (1), it took an additional 117 years to elucidate a second mechanism by which neutrophils can entrap microbial agents (2). In 2004, Brinkmann and colleagues reported that neutrophils can release nuclear chromatin together with granule proteins to form an extracellular mesh that binds and kills bacteria while also degrading virulence factors (2). The term neutrophil extracellular traps (NETs) was introduced to describe these neutrophil-derived antimicrobial fibers (2). Since the extracellular release of chromatin has historically been linked to necrosis (3), these initial findings allowed for speculation as to whether NETs were released from necrotic cells or as an active function of live neutrophils (4). It was later shown that NETs are released as a consequence of a regulated form of cell death called NETosis, which is dependent on the generation of reactive oxygen species (ROS) by NADPH oxidase (5, 6). To date, NETs and NETosis are used synonymously.

The discovery of NETs has generated an avalanche of research over the past 10 years, providing important insights into novel antimicrobial pathways and a plethora of pathologies as diverse as sepsis and thrombosis, autoimmune and metabolic diseases, and immunodeficiency and cancer. At the same time, the definition of NETosis has evolved rapidly, suffering many modifications that allowed researchers to incorporate and potentially misclassify almost any biological process involving the extrusion of neutrophil DNA (nuclear or mitochondrial) (7). The term “extracellular trap” has moreover been applied to an increasing number of cell types that release chromatin (e.g., eosinophils, mast cells, and macrophages) (8).

While the initiation and execution components during NETosis are not yet fully understood, unique cellular changes and biochemical pathways that distinguish NETosis from other forms of cell death have been well characterized (5, 9–11). Despite this, the definition of NETs/NETosis still hinges on purely morphological descriptors, primarily the extrusion of DNA. This arguably broad definition has generated an ever-increasing list of stimuli and pathways reported to induce NETs, while NETosis has inevitably become an umbrella term that encompasses a diverse group of cellular processes with potentially fundamental differences in biology and significance for immunity and disease. While some NETs are part of a unique form of programed cell death that evolved to fight pathogens (referred to in this review as NETosis), it is conceivable that many processes currently identified as “NETosis” may be the consequence of cellular damage or other forms of cell death (12). Distinguishing these different forms of NETs has critical implications. If NETs are driven by different biological processes, it is unlikely that all forms of DNA extrusion will have the same capacity to fight infection, drive various human diseases, or be inhibited by targeting a single biochemical pathway.

Our interest in NETs is related to the process of protein citrullination. To date, it is commonly accepted that citrullination of histones by peptidylarginine deiminase type 4 (PAD4) is required for chromatin unfolding and the formation of NETs (13). This misconception has drawn attention to PAD4 as a promising cellular target to broadly inhibit NETosis (14). Additionally, this has led to the proposal that this form of neutrophil death represents a major source of citrullinated autoantigens in rheumatoid arthritis (RA) (15, 16), an autoimmune disease characterized by the appearance of anti-citrullinated protein antibodies (ACPAs) (17). Similarly, it has focused interest on PAD4 as a therapeutic target in systemic lupus erythematosus (SLE) in which NETosis is believed to play a pathogenic role (18).

In this review, we will revisit some aspects of citrullination during the process of “NETosis” that we consider controversial. We provide experimental evidence to highlight differences in protein citrullination induced by various NET-inducing stimuli and propose a framework to understand these disparities. The corollary of this work is that protein citrullination can distinguish at least two mutually exclusive mechanisms that generate extracellular DNA structures. One of these is NETosis, an antimicrobial process that is not associated with cellular hypercitrullination. The second mechanism, which merely mimics NETosis, is induced by calcium ionophores or membranolytic agents that cause calcium-dependent hyperactivation of PADs, cellular hypercitrullination, and as a consequence extrusion of DNA. This mechanism, which we call leukotoxic hypercitrullination (LTH), is not antimicrobial, but linked to the production of citrullinated antigens in RA. Moreover, it is utilized by some pathogenic bacteria to abrogate neutrophil activity. A third process that can readily be confused with NETosis is the extracellular release of mitochondrial DNA (mtDNA), which results from a constitutive defect in mitophagy in neutrophils (19). This process has not been associated with protein citrullination. However, it is linked to the production of extracellular oxidized mtDNA (ox-mtDNA), which is highly interferogenic and initially confounded with NETosis in SLE (19). The need for a biochemical definition of NETs and their distinction from other mechanisms of neutrophil damage based on their antimicrobial and pathogenic effects will be discussed.

### NETs Released during Antimicrobial Programed Cell Death (NETosis)

The capacity of neutrophils to die by ROS-dependent “autotoxicity” in response to phorbol myristate acetate (PMA), a cocarcinogen derived from oil of the *Croton* shrub (20), was initially described by Min-Fu Tsan in 1980 (21). It was later appreciated that this form of neutrophil death differs from apoptosis and necrosis. Early changes in nuclear morphology, specifically chromatin decondensation and rupture of the nuclear envelope, are followed by rupture of the plasma membrane and subsequent dispersal of cytoplasmic contents into the extracellular space (22). The significance of this novel form of cell death was not recognized until 2004, when Brinkmann and colleagues reported two major findings (2). First, they found that the material released from the dying neutrophils was chromatin coated with granular antimicrobial proteins, forming extracellular fibers with bacterial binding capacity (NETs). Second, they noted that these fibers have the capacity to degrade virulence factors and kill bacteria, suggesting a novel mechanism by which the innate immune system can limit acute infections. Importantly, the production of NETs was reproduced with two physiological stimuli: interleukin-8 (IL-8) and lipopolysaccharide (LPS). The existence of NETs *in vivo* was confirmed in both experimental dysentery and spontaneous human appendicitis (2). These studies thus provided evidence that NETs may not simply be an artifact of PMA toxicity, but rather a process of potential physiological relevance.

Several features initially suggested that NETs were actively generated by neutrophils and not just a consequence of cellular rupture resulting from known forms of cell death (2). The concept that live neutrophils can actively weave extracellular traps using their own DNA created substantial excitement about this novel antibacterial process. Yet, in the absence of molecular mechanism, the argument that NETs were merely an artifact of necrosis remained (4). Subsequent studies demonstrated that NETs indeed emerge from dying neutrophils (5), but this process was neither related to neutrophil apoptosis nor necrosis. Instead, it was shown that PMA induces NETs through the unique form of cell death that was dependent on ROS production by NADPH oxidase (5, 21, 22). Acknowledging the non-accidental nature of committed cell death by NET formation distinct of necrosis, this process was named NETosis (in analogy to other forms of programed cell death) (6). Parallel studies demonstrated that *Staphylococcus aureus* (*S. aureus*) is able to replicate the changes in neutrophil morphology seen with PMA, supporting the biological relevance of this process as an antibacterial strategy. However, bacterial growth conditions that avoided expression of staphylococcal toxins (toxin-free *S. aureus*) were required to reproduce NETosis (5). The significance of using toxin-free, rather than virulent bacteria in these experiments will be discussed later.

### Morphological and Biochemical Features That Define NETosis

Neutrophil extracellular traps induced by PMA or “toxin-free” *S. aureus* exhibit unique features that support NETosis as a novel, regulated form of cell death (5). Morphologically, NETosis begins with the disappearance of nuclear lobules. This is followed by chromatin decondensation and disintegration of the nuclear envelope into small vesicles containing this decondensed chromatin. Subsequently, the membranes of these nuclear vesicles and cytoplasmic granules disintegrate, allowing for mixing of chromatin with cytoplasmic and granule contents. Finally, the plasma membrane ruptures and allows for the release of chromatin decorated with antimicrobial granule proteins into the extracellular space (the NET). While this process is distinct from necrosis and apoptosis (5), it is still uncertain whether NETosis may be mechanistically associated with other forms of regulated cell death (e.g., autophagy and necroptosis) (12, 23–27).

The production of ROS by NADPH oxidase is considered the biochemical hallmark in the process of NETosis (5). Much of the current understanding of the molecular mechanisms driving NETosis is based on studies using PMA, a phorbol ester that mimics diacylglycerol (20, 28). PMA directly activates protein kinase C (PKC) (28), which then phosphorylates the p40^phox^ and p47^phox^ components of NADPH oxidase. This induces the production of superoxide (29–31). Several kinases have been implicated downstream of PKC, including c-Raf, MEK, Akt, and ERK (11, 32–34). However, the mechanistic role of these enzymes is not well understood. For example, ERK has been proposed as a major downstream kinase involved in NETosis *via* phosphorylation of p47^phox^ and activation of NADPH oxidase (11). However, conflicting evidence exists as to whether ERK is indeed activated upstream or downstream of ROS production (11, 34). Nevertheless, an additional contribution of ERK to this process may not be required as PKC can directly activate NADPH oxidase (29–31).

Not every neutrophil undergoes NETosis upon activation with PMA. The mechanism behind why some cells survive and others die remains unclear. In cells that commit to NETosis, oxidative burst triggers the dissociation and activation of neutrophil elastase (NE) from a membrane-associated complex called the “azurosome” in a myeloperoxidase (MPO)-dependent process (35). Once in the cytoplasm, NE first cleaves F-actin and subsequently translocates to the nucleus. Here, NE degrades histones, thereby promoting chromatin decondensation (9, 10, 35). The degradation and disassembly of the actin cytoskeleton may further facilitate the disruption of the cytoplasmic membrane, a requirement for NET release (35). Citrullination of histones, along with their proteolytic cleavage, is considered essential for the disassembly of chromatin and release of DNA during NETosis (36, 37). Evidence for the presumed citrullination of histones during NETosis is reviewed in the next sections.

### PAD Activation and Cellular Hypercitrullination in Neutrophils

Citrulline is a non-essential amino acid that is generated in proteins by posttranslational enzymatic conversion of arginine residues to peptidylcitrulline. This modification, called either deimination or citrullination, is catalyzed by the PADs, a family of calcium-dependent enzymes (38–40). Among the five PAD enzymes encoded in humans (PAD1–PAD4 and PAD6) (41–45), PAD4 has received special attention in neutrophil biology. This enzyme, initially named PAD5 (PAD V), was cloned from a human myeloid leukemia cell line (HL-60 cells) induced to differentiate into granulocytes (44). Expression of PAD4 was later found in both human eosinophils and neutrophils (46). PAD4 shows nuclear localization, and histones were among the first PAD4 substrates to be identified (47, 48). To date, more than 70 putative PAD4 substrates have been described (49, 50). It was initially proposed that this enzyme may act as a transcriptional regulator by modifying the function of histones (51, 52), but PAD4-deficient mice demonstrated normal development, suggesting that, at least in mice, PAD4 has no essential role in steady-state neutrophil functions or development (37).

**Figure 1:**
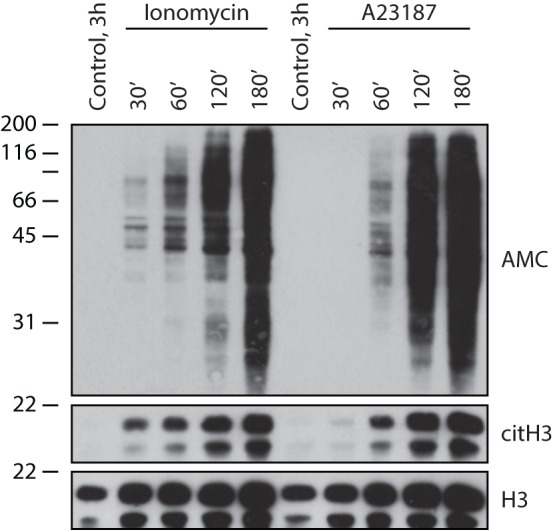
**Calcium ionophores induce cellular hypercitrullination in neutrophils**. After informed consent, peripheral blood neutrophils were isolated from healthy donors as previously described (58), resuspended in HBSS/10 mM Hepes/1.5 mM CaCl_2_, and incubated with 1 μM ionomycin or 5 μM A23187 at 37°C for 30–180 min. Neutrophils incubated for 3 h in the absence of stimulus were used as negative control. General protein citrullination was visualized by anti-modified citrulline (AMC) immunoblotting (59) (upper panel) and histone H3 citrullination (Cit-H3) using antibodies against citrullinated histone H3 (citrulline 2 + 8 + 17) (Abcam, Cat# ab5103) (middle panel). Histone H3 (H3) was detected as loading control (lower panel) (anti-histone H3, Abcam, Cat# ab1791). The experiments were performed on at least three separate occasions with similar results.

The mechanisms controlling PAD activation in cells are not yet fully understood. Since PADs are calcium-dependent enzymes (38), stimuli that potently increase intracellular calcium concentration have been used to identify cellular targets of protein citrullination in cells (48, 49, 53, 54). Ionophores are compounds that form lipid-soluble complexes with polar cations and act as vehicles in the transport of ions across biological membranes (55). A23187 (calcimycin) and ionomycin, both derived from *Streptomyces* species (55–57), are divalent carboxylic ionophores with selectivity for calcium (calcium ionophores). Induction of calcium influx by either A23187 or ionomycin in neutrophils results in hyperactivation of PADs and citrullination of a myriad of proteins across all molecular weights (48, 54), which we termed cellular hypercitrullination (58). During this process, citrullinated proteins accumulate with time and plateau ~3 h after cell activation (Figure 1).

Citrullination results in a loss of positive charge per modified arginine residue, thus reducing the net charge of the modified protein and increasing its hydrophobicity. This can lead to protein unfolding and either gain or more likely loss of protein function (51, 52, 60–62). While the spectrum of citrullinated proteins (i.e., the citrullinome) generated by calcium ionophores in neutrophils has not been fully characterized, known targets include nuclear proteins (nucleophosmin and histones) (48), antimicrobial proteins (MPO, NE, azurocidin, and defensins) (63), and components of the cytoskeleton (vimentin and actin) (54, 64). Among these substrates, citrullination of histones and vimentin promote chromatin decondensation and the disassembly of intermediate filaments, respectively (36, 61). The effect of hypercitrullination on other cellular proteins is unknown, but the vast accumulation of citrullination-induced protein unfolding is likely to provoke functional and structural collapse of the cell. For example, overexpression and spontaneous activation of PAD4 in osteosarcoma U2OS cells, without any other element required for NETosis (i.e., NE or MPO), is sufficient to induce cell disintegration and the production of NET-like structures (pseudo-NETosis) (65). This may also explain how neutrophils become dismantled and release their intracellular contents upon hyperactivation of PAD4 with calcium ionophores, including the release of genomic DNA (gDNA) that mimics NET formation (36). Although the mechanisms that drive cell damage and DNA extrusion by calcium ionophores are biochemically distinct from NETosis, as described in detail below (66), both PMA and calcium ionophores have been equivalently used to study this unique form of neutrophil cell death.

### The Misconception That PAD4 Is Required for NETosis

#### The Race to Find Mechanisms of Chromatin Decondensation during NETosis

While early studies demonstrated that ROS production by NADPH oxidase was required to initiate NET formation (5), the discovery of a downstream molecular mechanism driving chromatin decondensation during NETosis, specifically the cleavage of histones by NE, lagged behind (10, 35). During this time, it was found that (1) hypercitrullination of histones by PAD4 promotes chromatin unfolding and (2) calcium ionophores, potent activators of PADs, induce extracellular release of DNA similar to NETosis (36). Assuming that the nature of NETs generated by calcium ionophores and PMA were the same, PAD4 activation and histone citrullination were proposed to be the missing link between ROS production and chromatin decondensation during NETosis (13, 36). In fact, the belief that PAD4 is activated by ROS during NETosis prevails to this day (67), although evidence to support this concept is lacking. Since a reducing environment is necessary to maintain the active site free thiol Cys645 required for PAD4 function (68, 69), we can anticipate that oxidation of PAD4 by ROS will instead inactivate the enzyme.

**Figure 2:**
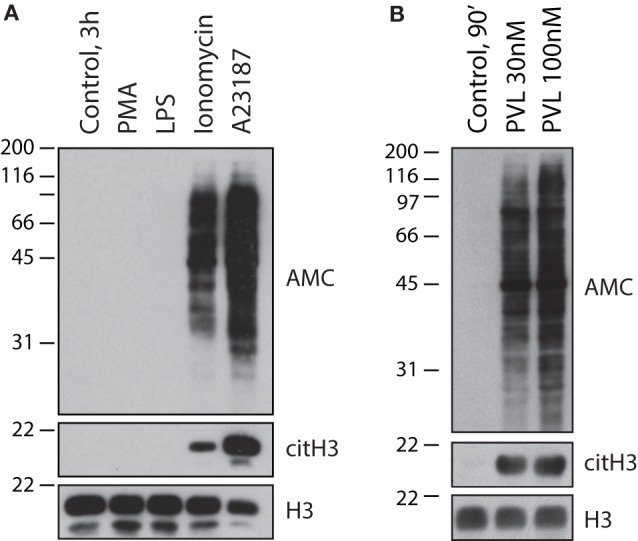
**Calcium ionophores and the pore-forming toxin PVL from *S. aureus*, but not NETosis-inducing stimuli, activate cellular hypercitrullination in neutrophils**. Purified human neutrophils in HBSS/10 mM Hepes/1.5 mM CaCl_2_ were incubated **(A)** in the absence or presence of 100 nM PMA, 500 ng/mL LPS, 1 μM ionomycin, or 5 μM A23187 at 37°C for 3 h, or **(B)** in the absence or presence of PLV from *S. aureus* (recombinant LukS and LukF from IBT BioServices) at 30 or 100 nM for 90 min at 37°C. Total citrullinated proteins (AMC), citrullinated histone H3 (citH3), and histone H3 (H3) (loading control) were detected by immunoblotting. The experiments were performed on at least two separate occasions with similar results.

While a lack of biochemical insight initially suggested that NETs induced by calcium ionophores and PMA were identical, subsequent studies established that they are the consequence of biologically distinct processes. The formation of NET-like structures induced by calcium ionophores occurs more rapidly than those formed during NETosis. Additionally, this process is independent of ERK and NADPH oxidase activity (a hallmark of NETosis), and thus often referred to as NADPH oxidase-independent NET formation (32, 66). These calcium ionophore-induced structures are in part mediated by calcium-activated small conductance potassium (SK) channel member SK3 and mitochondrial ROS (mtROS) (32), and completely dependent on calcium influx, unlike those formed during PMA-induced NETosis (32, 66). No evidence exists indicating that NE is required for the induction of “NETs” or citrullination by calcium ionophores (70), nor that F-actin and/or histones are cleaved during this process, as required during NETosis (10, 35). Notably, the calcium requirement for calcium ionophore-induced “NET” formation is not surprising as PAD activation and cellular hypercitrullination are calcium-dependent processes. In contrast to calcium ionophores, however, NETosis induced by PMA proceeds without PAD4 activation and histone citrullination (32, 71). Moreover, PMA is not only unable to initiate protein citrullination in neutrophils (Figure 2A), but also acts as a potent inhibitor of PAD4 in these cells (71). As such, PAD4 hyperactivation induced with calcium ionophores is significantly inhibited by PMA in a process that is dependent on PKCα (71). While the mechanism by which this kinase suppresses the activity of PAD4 is not elucidated, it is interesting that a different PKC isoform (i.e., PKCζ) is required for PAD4 activation induced by calcium ionophores (71). This suggests that PAD4 is regulated *via* phosphorylation. By activating different kinases, PAD4 function may be stimulus dependent, thus avoiding unwanted effects of protein citrullination during NETosis and other biologically relevant processes.

Two major conclusions can be drawn from these data. First, calcium ionophores can induce cellular hypercitrullination and NET-like structures, but this process is not associated with NETosis (i.e., an antimicrobial form of cell death dependent on NADPH oxidase) (5). Second, NETosis is associated with a decrease in PAD4 activity and absence of cellular hypercitrullination (Figure 2A). Regrettably, the original misconception that hypercitrullination occurs during NETosis remains to date.

#### The Erroneous Use of Citrullinated Histones as Specific Markers of NETosis

The identification of biochemical markers associated with specific forms of cell death is critical to study their mechanisms and biological relevance both *in vitro* and *in vivo* (23). Different to other forms of programed cell death, unique markers of NETosis have not yet been identified. Although cellular hypercitrullination induced by calcium ionophores targets a broad range of proteins (48, 54), initial studies using these ionophores to suggest a role of citrullination during NETosis focused entirely on the analysis of histones (36). This erroneously created the idea that histones were the only targets of ionophore-induced citrullination in neutrophils, and that histone citrullination was the mechanism underlying dismantling of the cell in response to calcium influx. Not only did the misconception that calcium ionophores induce NETosis become accepted, but it also fueled dogmatic belief that histone citrullination can serve as a specific biomarker for this form of cell death. Given that PAD4 is actively inhibited during NETosis (71), the detection of citrullinated histones both *in vitro* and *in vivo* can be explained by other means.

First, PAD4 targets histones as a mechanism to regulate transcription upon cell activation (72–74). The detection of citrullinated histones alone *in vivo* may thus identify activated cells, rather than cells dying by NETosis (75). Second, histone citrullination is similarly observed in other forms of cell death such as apoptosis (58, 76), while shown to be inhibited during NETosis (71). Identifying extracellular citrullinated histones together with other neutrophil contents (DNA, MPO, PR3, and elastase, among others) is therefore not a suitable indicator of NETosis, but may rather indicate that neutrophils are dying by a form of cell death that is distinct from NETosis. Third, prominent citrullination of histones and extracellular release of DNA are rapidly induced as consequence of membranolytic damage of neutrophils (i.e., LTH). This distinct form of death can easily be confused with NETosis, as discussed in detail below. Finally, isolated neutrophils, in the absence of NETosis-inducing stimuli, show increased staining for citrullinated histones over time. This may represent spontaneous cell death by apoptosis or evolving activation/damage suffered during neutrophil purification. In the absence of appropriate negative controls, any stimulus, including PMA, will thus appear to induce neutrophil citrullination. Importantly, the use of PAD4-deficient cells or PAD inhibitors is not suitable negative controls to discard spontaneous activation of PAD4. In the opinion of these authors, a large number of studies that have reported citrullination in the setting of NETosis lack appropriate controls to delineate whether citrullination is induced upon stimulation or merely represents background staining. The absence of negative controls may thus explain why an unlikely number of stimuli tested have been shown to induce some degree of citrullination in neutrophils, and why the reproducibility of these findings remains poor.

#### The Haste of Linking NETosis and Citrullination

The excitement and expectations regarding citrullination and NETosis for systemic autoimmunity, the arbitrary use of PMA and calcium ionophores to induce NETs, and some overzealous data interpretation may have also contributed to keep the erroneous concepts about hypercitrullination in NETosis alive. For example, in a frequently cited review on PAD4 and NET formation (13), 16 references were included to establish that PMA induces PAD4 activation during NETosis. This includes the original paper by Brinkmann and colleagues, which was published 5 years prior to data suggesting that PAD4 is involved in NET formation (2, 9, 10, 27, 37, 77–87). In fact, only 1 of these 16 references included experiments involving protein citrullination. The majority of the referenced studies merely noted the presence of histones in NETs, and not histone citrullination in NETs.

The misconception that PMA induces hypercitrullination and that calcium ionophores drive NETosis is more than a semantic problem. For example, in a recent study, NETs induced with PMA were used to demonstrate that monoclonal antibodies generated from RA synovial B cells target citrullinated histones in NETs (88). Considering that PMA effectively blocks citrullination (71), it is much more likely that cross-reactivity of RA autoantibodies with native (uncitrullinated) histones or other NET components explains these findings. Nevertheless, the study embraced the misconception that NETosis is a source of citrullinated autoantigens in RA. Similarly, calcium ionophores are potent inducers of neutrophil hypercitrullination and citrullinated RA autoantigens (54, 63), which may be erroneously interpreted as the result of NETosis. In this context, the premature excitement and hastiness surrounding the rapidly growing field of NETosis has already misguided our understanding of etiology and pathogenesis of human diseases.

#### The Incomplete Understanding of the Role of PAD4 in the Immune System

Peptidylarginine deiminase type 4 plays a major role in epigenetic regulation *via* the citrullination of histones and transcription factors (72–74, 89–95). In the immune system, PAD4 promotes cytokine production by augmenting chromatin association of E2F-1 and decreasing HP1-mediated transcriptional repression of cytokine genes (74, 89). Moreover, PAD4 inhibitors were shown to decrease pro-inflammatory Th1 and Th17 responses and to increase anti-inflammatory Th2 responses both *in vitro* and *in vivo* (96). PAD inhibition also blocks the functional maturation of dendritic cells induced by toll-like receptor agonists (97). Despite this multifaceted role of PAD4 for immune function, the prominent misconception that PAD4 is required for NETosis has frequently led investigators to postulate that the anti-inflammatory effects of PAD inhibitors seen *in vivo* are best explained by blocking NET formation. Regardless of whether NETosis may have any role in disease pathogenesis, the observed benefit of PAD inhibition in experimental models of inflammation and autoimmunity is thus haphazardly attributed to limiting NET formation. This has importantly led to the current paradigm that NETs are pathogenic *in vivo*, confounding our understanding of disease pathogenesis.

For example, inhibition of NETosis by propylthiouracil (PTU) induces the production of anti-MPO antibodies and vasculitis in rats (98), suggesting that the normal process of NETosis is protective against this autoimmune process. However, the finding that PAD inhibition by Cl-amidine decreases PTU-induced autoantibody production in this model has been paradoxically interpreted as evidence that excessive formation of NETs is implicated in the production of these antibodies and vasculitis (75). The possibility that PAD inhibition may act through mechanisms different from NETosis was not considered. In the case of SLE, cumulative evidence now suggests that NETosis is not involved in the disease pathogenesis, as discussed below (19, 99, 100). Thus, the demonstrated benefits of PAD inhibition in experimental models of lupus, attributed to a decreased burden of NETosis (18, 101), may indeed be mediated by alternative mechanisms, such as the transcriptional regulation of anti-inflammatory pathways by these inhibitors. The inhibition of PAD4 activity as direct evidence *in vivo* that citrullination and NETosis are linked to disease pathogenesis therefore requires critical evaluation.

### PMA-Induced NETosis vs. NETs Induced by Calcium Ionophores: Biological Relevance and Proposed Physiological Counterparts

Phorbol myristate acetate and calcium ionophores have had a historical role in the functional understanding of the immune system. Since the early 1970s, these molecules have been used as surrogate stimuli that mimic signals induced by microbial and immune products, simplifying the study of mechanisms and pathways of immune activation (102, 103). In neutrophils, PMA potently activates ROS production *via* NADPH oxidase and induces degranulation (104–109). PMA has no effect on intracellular calcium during neutrophil activation (110, 111). This may also explain the inability of PMA to activate PAD4, in addition to the inhibition mediated by PKCα (71). In contrast, calcium ionophores have been used to study the role of calcium in neutrophil function (109, 112–114). While calcium ionophores also induce neutrophil degranulation (109, 114), these molecules are extremely weak stimulators of NADPH oxidase activity as compared to PMA (110, 115). Because of their functional divergence, PMA and calcium ionophores are commonly used in combination to achieve maximal activation of cellular responses that require elevated intracellular calcium and PKC activation (116, 117).

Although a large number of stimuli with potential biological relevance have been proposed to induce NETosis (13, 118), the underlying mechanisms for the majority of these stimuli have not been elucidated. Instead, studies have focused on understanding the pathways used by PMA and calcium ionophores to induce NETs, assuming that these surrogates are analogous to NETosis *in vivo*. The biochemical pathways activated by immune and microbial stimuli, however, may not completely replicate those induced by PMA or calcium ionophores. Stimuli such as IL-8 and LPS, initially used to demonstrate that NETs are not simply an artifact of PMA treatment, are neither efficient in driving ROS production (as seen with PMA) nor in increasing intracellular calcium (analogous to calcium ionophores) (119–123). The precise mechanism of NET induction with both IL-8 and LPS is unknown, and their ability to reliably reproduce NETosis is questionable (27, 124). Other soluble factors, such as tumor necrosis factor-α (TNF-α) (37, 79), have similar limitations in explaining the induction of NETs (125).

The potential importance of NETs in human biology is supported by the direct induction of NETosis by pathogens. The fungi *Candida albicans* (*C. albicans*) and *Aspergillus fumigatus* (*A. fumigatus*) appear to be ideal model pathogens for consistently activating NETosis *via* NADPH oxidase, MPO, and NE, thus using a mechanism similar to PMA (9, 10, 35, 126–129). However, there is no evidence that citrullination or pathways activated by calcium ionophores are required by these fungi to induce NETosis.

The mechanisms of bacterial induction of NETs, however, are inconsistent. *S. aureus* has been used as a model organism to demonstrate bacterial NET formation by both NADPH oxidase-dependent (PMA-like) and NADPH oxidase-independent pathways (5, 81). *S. aureus* has also been reported to induce “vital” NET formation, a morphological description chosen to highlight that neutrophils remain “alive” (i.e., transiently functional and somewhat motile) after releasing NET-like structures (130, 131). Other bacterial species face similar caveats. For example, *Klebsiella pneumoniae* has been shown to require MPO and NE to induce NETosis in mice (PMA-like?) (10). However, the induction of NETs by *K. pneumoniae in vitro* was not reproducible (128). In the case of *Shigella flexneri* (*S. flexneri*) and group A *Streptococcus pyogenes* (*S. pyogenes*) deficient in an extracellular DNase (Sda1), used to support that PAD4 is required to induce NETs (37), the mechanism(s) that may drive PAD4 activation remain elusive. Indeed, it has been proposed that only large pathogens such as *C. albicans* hyphae and bacterial aggregates have the capacity to induce NETosis, questioning experiments that involve the induction of NETosis by single-cell suspensions of bacteria that are not aggregated (128).

The poor reproducibility of NET formation by some biologically relevant stimuli and the limited understanding of these discrepancies may explain why PMA and calcium ionophores remain primary tools in the study of NETs.

#### Synergistic Signals May Be Required to Efficiently Trigger NADPH Oxidase-Dependent NETosis

Several stimuli that were initially proposed to induce NETs are poor activators of NADPH oxidase (such as IL-8, LPS, and TNF-α) (119–123, 125). However, it is fascinating that these molecules can prime neutrophils to produce high amounts of ROS in response to a second signal, such as fMLP (119, 120, 125), a peptide that mimics bacterially derived signals by activating the formyl peptide receptor 1 (FPR1) (132). Similarly, neutrophil priming with interleukin-1β (IL-1β), TNF-α, granulocyte macrophage colony-stimulating factor (GM-CSF), and granulocyte colony-stimulating factor (G-CSF) enhances ROS production by IL-8 (121, 123). Although the ability of LPS itself to induce NETs is uncertain, the combination of platelets and LPS induces platelet aggregation and acts as a potent stimulus for NETosis (124). Therefore, it is conceivable that NETs are induced under synergistic circumstances where more than one inflammatory and/or bacterial stimulus may be present (e.g., infection), although these stimuli in isolation may not induce NETosis.

#### NET-Like Structures Induced by Calcium Ionophores Mimic Damage to the Cellular Membrane

Stimuli that induce NETs through physiological pathways mimicking the action of calcium ionophores are more difficult to elucidate. PADs are calcium-dependent enzymes that require millimolar amounts of calcium for *in vitro* activity (38). In cells, however, PAD activation can be observed during biological processes (73) and in response to physiologic stimuli (51, 93, 94, 133) that do not increase intracellular calcium concentrations above the nanomolar range. Activation of PADs has therefore been posited to require cellular cofactors that modulate calcium sensitivity of the enzyme. Nonetheless, citrullination under these conditions represents a tightly controlled process. Protein citrullination appears limited to nuclear substrates involved in gene regulation such as histones and transcription factors, with no damage to the cell (51, 73, 93, 94, 133). In contrast, calcium ionophores induce hypercitrullination of proteins across multiple cellular compartments, including many likely “accidental” targets. These fundamental differences between limited protein citrullination induced by physiologic stimuli and hypercitrullination observed with calcium ionophores may be best explained by the kinetics and absolute amount of calcium influx.

Calcium ionophores induce rapid, prominent, and sustained increases in cytosolic calcium concentration in neutrophils (above 1 μM) (110, 111). In contrast, even the most potent physiologic stimuli only induce transient increases in cytosolic calcium below 1 μM. fMLP, likely one of the most potent activators of intracellular calcium signaling, increases cytosolic calcium to a transient peak of 500–800 nM that returns to baseline within 2–3 min (111, 134, 135). Other stimuli, such as IL-8 and C5a, generate similar transient increases in cytosolic calcium, but with much smaller effect sizes (~300–400 nM) (110, 123). Under these conditions, it is hard to envision that PAD4 could be hyperactivated by these stimuli. Indeed, fMLP, IL-8, and other stimuli that induce transient cytosolic calcium increases are unable to reproduce calcium ionophore-induced hypercitrullination (58). Moreover, fMLP fails to induce NETs (27), reinforcing that limited elevations in intracellular calcium are insufficient to generate NET-like structures. In contrast to calcium ionophores, which lead to decreased chemotaxis (likely because the cells disintegrate) (113, 136), intracellular calcium signals activated by fMLP, IL-8, C5a, and interferon (IFN)-γ are potent chemotactic factors (122, 137–139). This again highlights major physiological differences between signals that slightly increase cytosolic calcium and the effect of calcium ionophores.

Considering that no physiological stimulus is known to reproduce the effects of calcium ionophores on neutrophils, we propose that ROS production *via* NADPH oxidase is the primary mechanism for NETosis. In contrast, NET-like structures induced by calcium ionophores (a form of cell death distinct from NETosis) may be representative of pathological conditions that drive massive intracellular calcium influx and hypercitrullination as result of direct damage to the cellular membrane. To distinguish this form of neutrophil death from NETosis, we will use the term leukotoxic hypercitrullination.

### Bacterial and Immune Pore-Forming Proteins Induce Neutrophil Death by Leukotoxic Hypercitrullination

Several unique features distinguish LTH from NETosis, which are as follows: (1) the NET-like structures are triggered by prominent and sustained calcium influx and can be inhibited by chelation of extracellular calcium (66), (2) are generated independently of NADPH oxidase activity (66), (3) require PAD4 activity and are suppressed by PAD inhibitors (14, 36), (4) undergo rapid formation (within minutes) (36), and (5) protein citrullination is not limited to histones and transcription factors, but encompassing proteins across all molecular weights (54). Whether cellular membrane rupture is required for the release of nuclear content into the extracellular space during LTH is not known.

#### *S. aureus*, Staphylococcal Pore-Forming Toxins, and Leukotoxic Hypercitrullination during Bacterial Infection

The idea that NET-like structures induced by calcium ionophores are representative of membranolytic pathways is derived from the studies of *S. aureus*, which induces NETs through a variety of mechanisms (5, 81, 130). To induce bactericidal (true) NETosis, *S. aureus* requires growth under conditions that preclude the expression of bacterial toxins (5). In contrast, when neutrophils are exposed to toxin-producing strains of *S. aureus*, rapid and NADPH oxidase-independent formation of NET-like structures was observed (81). Strikingly, the study also identified that this unique form of “NETosis” (we argue this actually represents LTH) was triggered by Panton–Valentine leucocidin (PVL), a pore-forming toxin secreted by highly virulent *S. aureus* strains that induces a rapid, prominent, and sustained increase in intracellular calcium (140, 141). Since phagocytosis is a potent stimulus to activate NADPH oxidase (142), it is likely that, by avoiding the action of pore-forming toxins, the engulfment of *S. aureus* by neutrophils in the initial studies by Fuchs and colleagues allowed for ROS production and NETosis (5). However, LTH (toxin-induced neutrophil death without phagocytosis) is likely to predominate during human bacterial infection. This mirrors the initial morphological observations of dying neutrophils during human skin infection by Metchnikoff (1). Interestingly, different to NETosis that requires rupture of the cellular membrane to release DNA and NETs, NET formation induced by PVL appears to release chromatin *via* nuclear envelope blebbing and vesicular exportation, preserving the integrity of the cell membrane (81). In fact, “vital NETosis” described in mice infected with *S. aureus* may represent LTH induced by PVL or other staphylococcal pore-forming toxins (130, 131). In addition to PVL, *S. aureus* produces several other membranolytic toxins that can induce rapid formation of NET-like structures (143, 144). These proteins include N-terminal ArgD peptides and leukotoxin GH (LukGH), possibly among others that have not been studied in detail.

Finally, two additional features are important to support that membrane pore formation, calcium influx, and hypercitrullination are involved in the process of chromatin extrusion induced by *S. aureus*. First, it has been demonstrated that PAD4 inhibitors decrease the production of “NETs” by *S. aureus* (14). Second, we demonstrate here that PVL is a potent inducer of cellular hypercitrullination in human neutrophils (Figure 2B), which contrast with the absence of citrullination in neutrophils activated with PMA (Figure 2A). Thus, LTH, but not NETosis, is likely the mechanism underlying NADPH oxidase-independent “NET” formation observed with *S. aureus*.

#### Bacterial Pore-Forming Toxins as Triggers of Leukotoxic Hypercitrullination – The Need for Standards in the Definition and Study of NETs

The model that bacterial pore-forming proteins are responsible for inducing LTH and secondary extrusion of nuclear material is not limited to *S. aureus*. Pore-forming toxins are potent virulence factors produced by many pathogenic bacteria carrying diverse cellular specificities (145–147). These protein toxins have the ability to change from a water-soluble state to a membrane-bound and membrane-inserted conformation. This large family of molecules includes both short peptides and large globular proteins and is expressed both by Gram-positive and Gram-negative bacterial species alike (145–147).

For the context of this review, the primary and unifying effect of bacterial pore-forming toxins is the ability to induce rapid changes in ion concentration in the cytosol of their respective target cells, specifically an increase in intracellular calcium (145, 148). Early studies on the mechanisms of cell damage by bacterial pore-forming toxins indeed suggested that these molecules act as calcium ionophores (149). The capacity of toxins secreted by bacteria to induce NET-like structures is not novel and has previously gained the attention of others (150). However, the potential link between bacterial pore formation, calcium influx, and hypercitrullination has not been considered as a driver of cellular damage and DNA extrusion. The production of pore-forming toxins therefore is a critical variable that must be considered to interpret mechanisms of NET formation used by pathogenic bacteria. It is thus important to communicate whether bacteria were cultured under conditions that allow for toxin production, whether the specific strains used produce or are deficient of toxins, the spectrum of toxin expression, and their concentration. Finally, future studies on NETosis should be required to disclose whether bacteria were opsonized to facilitate phagocytosis, as this may alter their ability to generate ROS and thus induce NETosis.

#### Inhibition of PAD4 Activity in NETosis and Leukotoxic Hypercitrullination

The use of PAD4-deficient mice and PAD4 inhibitors to block NET formation is frequently presented as evidence that citrullination is required for the induction of NETosis (14, 37). It is important to appreciate, however, that these studies employed conditions that promote PAD activation and DNA extrusion by calcium influx, not through stimuli that induce NETosis. In the case of PAD4 inhibitors (14), ionomycin and *S. aureus*, both inducers of calcium-dependent and NADPH oxidase-independent pathways, were used to demonstrate that PAD4 inhibition can block histone H3 citrullination and possibly the formation of “NETs” (albeit incorrectly quantified by histone H3 citrullination, not chromatin extrusion). The inhibition of “NETs” in a murine model of sepsis can be similarly explained by blocking LTH rather than NETosis (151).

In mice deficient in PAD4, *S. flexneri* and *S. pyogenes* were used to address the role of PAD4 activity during NETosis (37). Interestingly, both bacteria produce virulence factors that can affect calcium homeostasis. *Shigella* has been shown to induce calcium responses that are dependent on the type III secretory apparatus. This allows for the insertion of a pore containing the IpaB and IpaC proteins into host cell membranes and acts in conjunction with the pore-forming toxin hemolysin E (HlyE) (152–154). *S. pyogenes* streptolysin O (SLO), a cholesterol-dependent cytolysin (CDC), forms large transmembrane pores evolutionary related to immune pore-forming proteins that permit extracellular calcium flux into the target cell cytosol (147, 155).

While the abnormal activation of PAD4 induced by ionomycin or bacterial toxins can easily explain the effect of PAD4 inhibitors and PAD4 deficiency in these studies, it remains uncertain how these inhibitors could block PMA-induced NETosis (18), which proceeds without PAD4 activation (32, 71). It is conceivable that molecules designed to block PADs exert additional functions that indirectly affect PMA-induced NETosis. We also cannot exclude that some residual activity of PAD4 is maintained in the presence of PMA, resulting in limited citrullination of histones that may contribute to chromatin decondensation, without affecting their antimicrobial properties (as discussed below). Indeed, trace citrullination of histone H3 has been reported in neutrophils in response to LPS and other stimuli (71, 156). However, some of these stimuli are highly unreliable means to induce NETosis (27, 124).

Since the expression of pore-forming toxins easily confounds the study of NETosis induced by bacterial species, the fungi *C. albicans* and *A. fumigatus* may represent a better model to interrogate the effect of PAD4 deficiency and inhibition in the induction of NETs.

#### Immune Pore-Forming Proteins – A Role for Complement and Perforin in Leukotoxic Hypercitrullination?

**Figure 3:**
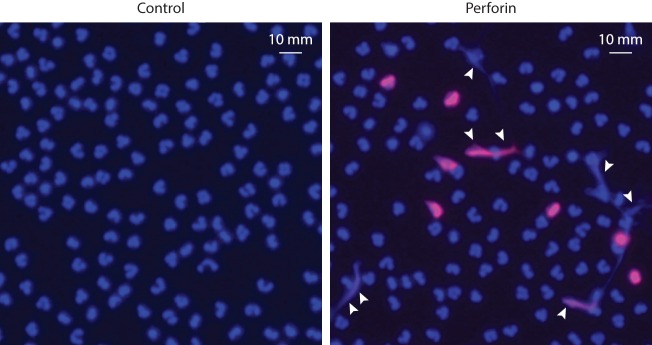
**Purified perforin induces PAD activation and the release of extracellular DNA in neutrophils**. Human neutrophils (5 × 10^5^ cells/50 μL) in HBSS/10 mM Hepes/1.5 mM CaCl_2_ were plated onto standard microscope slides coated with poly-d-lysine (Sigma) and incubated for 30 min at 37°C to allow for cell attachment. Neutrophils were then incubated in the absence or presence of sublytic amounts (500 ng/mL) of purified perforin (Enzo Life Science) for 1 h at 37°C, as previously described (58). Then, the cells were fixed, permeabilized, and stained with anti-histone H3 (citrulline 2 + 8 + 17) antibodies (H3cit) and Alexa Fluor 594 goat anti-rabbit F(ab′)_2_ fragment (Invitrogen, Cat# A-11072). Cells were mounted with ProLong Gold (Molecular Probes) plus DAPI. Samples were analyzed using a Zeiss axioscope microscope. Excitation filter: 510–560 nm for Alexa Fluor 594 and excitation filter: 300–390 nm for DAPI. Merged images of DAPI (blue) and H3cit (red) staining are shown. Perforin induces PAD activation (detected by citrullination of histone H3) and the extracellular release of neutrophil DNA (arrowheads). The experiments were performed on at least four separated occasions, with similar results.

Pore-forming proteins are not exclusive to microbial species. Vertebrates have evolved similar systems to kill infectious agents [i.e., the complement membrane attack complex (MAC)], as well as infected or malignant cells (i.e., perforin) (157). Similar to other proteins that create transmembrane pores, MAC and perforin promote prominent calcium influx, PAD activation, and cellular hypercitrullination (58). Moreover, we found that perforin also induces the extracellular release of DNA (Figure 3), which may erroneously be attributed to NETosis in the future. We anticipate that killing of neutrophils by complement MAC would have a similar effect.

We believe that these data are consistent with the herein proposed model that calcium ionophores act analogous to pore-forming proteins, which drive cellular damage, hypercitrullination, and chromatin extrusion mimicking NETosis (i.e., LTH). Defining the independent role of toxins secreted by each pathogen shown to induce “NETosis” would be necessary to confirm or discard this hypothesis.

#### Does PAD2 Play a Role in Leukotoxic Hypercitrullination Induced by Pore-Forming Proteins?

PAD2 is an abundant enzyme in neutrophils and is potently activated in cells using calcium ionophores or perforin. Hypercitrullination induced by PAD2 generates a repertoire of citrullinated proteins that is even more prominent than the pattern observed with PAD4 (54, 58), with protein targets that are common and unique for both enzymes (50). Proteins of the cellular cytoskeleton, such as actin and vimentin, are prominently citrullinated by PAD2 (50, 54). This may contribute to the dismantling of neutrophils during cellular hypercitrullination. Whether hyperactivation of PAD2 plays any direct role in this process, as demonstrated for PAD4 (65), is unknown. Elucidating a potential role of PAD2 during LTH is of great interest as targeted inhibition of PAD4 will likely have negligible effects on PAD2 hyperactivation and its consequences.

### NETosis and Leukotoxic Hypercitrullination in Immunity: Protein Citrullination May Impair the Bactericidal Function of NETs

Based on our proposed model, neutrophils can suffer two biochemically distinct forms of cell death that can easily be confused morphologically. One is driven by signals that induce ROS production by NADPH oxidase (NETosis), the other involves bacterial toxins that generate transmembrane pores, abnormal calcium influx, neutrophil hypercitrullination, and the extracellular release of chromatin (LTH). Applying this model, what would be the biological significance of these distinct forms of neutrophil death?

In 1942, Miller and colleagues identified that histones have potent antimicrobial activity (158). It was later demonstrated that the arginine-rich histone fraction can effectively kill both Gram-positive and Gram-negative bacteria at nanomolar concentrations (159). More recently, the importance of histones as potent bactericidal factors was resurrected by the discovery that the antibacterial activity in NETs is largely dependent on histones (2). The mechanism by which histones kill bacteria is not fully understood, but has been attributed to their high content of arginine and lysine. These cationic residues are thought to allow for binding, disruption of the bacterial membrane, and translocation of small antibacterial molecules (160). Using histone-derived antimicrobial peptides, it was shown that arginine is required for antimicrobial activity, membrane embedding, and peptide translocation (160). Since histones are potent antimicrobial factors in NETs and their arginine content is critical for bactericidal function, it is not surprising that decreasing the number of arginine residues during histones citrullination reduces their potent antimicrobial activity (37). Similarly, citrullination of the cationic bactericidal protein LL37 attenuates its capacity to neutralize LPS and to protect against endotoxin-induced death in mice (161).

From an evolutionary perspective, we believe that the inactivation of PAD4 that occurs during NETosis is unlikely to be accidental (71). This inhibition may have evolved to avoid citrullination-mediated inactivation of critical antimicrobial factors (e.g., arginine-rich bactericidal proteins) released during NETosis. Although the spectrum of citrullinated proteins induced by bacterial toxins in neutrophils has not been described to date, antimicrobial proteins including MPO, NE, azurocidin, and defensins appear to be targets of citrullination induced by ionomycin (63). This suggests that antimicrobial components of NETs other than histones may be similarly inactivated by citrullination.

**Figure 4:**
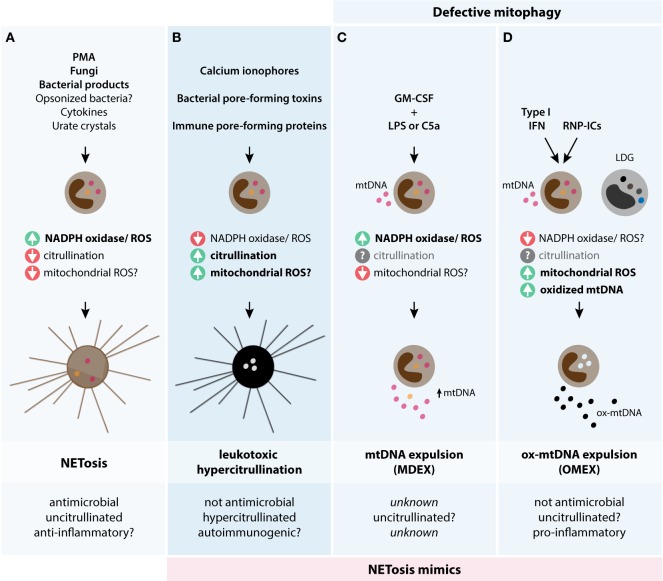
**Theoretical models of biological mechanisms of DNA release in neutrophils**. The three models are depicted and illustrate their potential triggers, biochemical pathways, and biological consequences. **(A)** NETosis is initiated by stimuli that potently activate NADPH oxidase and ROS production, but decreases PAD4 activation and likely mtROS production. NETosis is antimicrobial, potentially anti-inflammatory, and has limited protein citrullination. **(B)** Leukotoxic hypercitrullination is triggered by stimuli that generate prominent and sustained increases in cytosolic calcium, such as ionophores and proteins that damage the cellular membrane (e.g., pore-forming proteins). PAD hyperactivation and protein hypercitrullination are characteristic of this process, but not ROS production by NADPH oxidase. While mtROS may have some role in the release of DNA by calcium ionophores in neutrophils (32), the relevance of this pathway in neutrophil damage induced by pore-forming proteins is unknown. Antimicrobial functions and the immunogenicity of self-antigens are affected as a result of cellular hypercitrullination. **(C,D)** To compensate a constitutive defect in mitophagy, neutrophils normally extrude mitochondrial components, including mtDNA, as a mechanism for mitochondria clearance. **(C)** The process of mtDNA expulsion (MDEX) may be enhanced by inflammatory signals such LPS or C5a following neutrophil priming with GM-CSF and requires NADPH oxidase activity. **(D)** IFN-I and RNP-ICs alter the normal disposal of mitochondria in neutrophils and promote the expulsion of ox-mtDNA (OMEX). This process is dependent on mtROS, but not NADPH oxidase, and may be predisposed by factors that decrease NADPH oxidase activity. Different to mtDNA, ox-mtDNA is pro-inflammatory and highly interferogenic. LDGs from patients with SLE have spontaneous OMEX. The role of citrullination in **(C,D)** is unknown. The question marks (?) reflect pathways/functions that require further experimental confirmation.

NETosis and toxin-mediated LTH may represent two fundamentally opposed strategies used by the innate immune system and microbial pathogens to kill each other (Figure 4). NETosis defines a form of programed, antimicrobial cell death initiated by signaling pathways that suppress PAD activation and induce the release of bactericidal NETs. This process is dependent on ROS production *via* NADPH oxidase. Stimuli that generate NETosis may include inflammatory cytokines acting synergistically, bacterial products, fungi, and opsonized bacteria that can potently activate NADPH oxidase. In contrast, pathogens have evolved strategies that generate abnormal and sustained increases in cytosolic calcium of target cells through pore-forming toxins. This process hyperactivates calcium-dependent pathways (PADs), thus rapidly inactivating antimicrobial neutrophil factors and triggering disintegration of the target cell.

The deleterious effect of hyperactivation of PAD4 and LTH on the antimicrobial function of neutrophils is further supported by several findings, which are as follows: (1) NET-like structures induced by toxin-expressing strains of *S. aureus* have limited proteolytic activity; this loss of function is PVL-dependent (81), (2) NET-like structures induced by the *S. aureus* pore-forming toxin LukGH demonstrate no antimicrobial potential against this bacterium (144), in striking contrast to the potent bactericidal activity of PMA-induced NETs (2), (3) Group B *Streptococcus* induces neutrophil lysis, NET-like structures and resistance to NET-mediated bacterial killing via its hemolytic pigment/lipid toxin (162), (4) PAD4-deficient mice are partially protected from LPS-induced shock and demonstrate increased production of IL-10 (163), and (5) PAD inhibition increases survival in a murine model of sepsis (151), supporting that PAD4 activity negatively affects the host during infection.

### Leukotoxic Hypercitrullination vs. NETosis in Rheumatoid Arthritis Pathogenesis

The induction of LTH by pore-forming proteins may not only be detrimental for the clearance of pathogens. The hypercitrullination of self-proteins that are not physiological targets of PADs may have a primary role in generating neoantigens. This cellular dysregulation may have important implications for the etiology and pathogenesis of RA, as citrullinated proteins are major targets of the autoimmune response in this disease (17). Immune-mediated membranolytic pathways (perforin and MAC formation) are activated in the rheumatoid joint and may drive the generation of citrullinated autoantigens in established disease (58). Similarly, chronic hypercitrullination induced by bacterial pore-forming toxins may represent a primary source of citrullinated autoantigens in susceptible individuals with the potential to initiate the ACPA response and RA.

The hypothesis that NETosis is the source of citrullinated autoantigens in RA, as proposed in recent years, contradicts experimental data showing that hypercitrullination does not occur during NETosis (58, 71). This contradiction may be explained by the following points. (1) Studies on RA pathogenesis frequently use calcium ionophores to induce citrullination in neutrophils, which mimic LTH induced by pore-forming pathways, but not NETosis (63, 164). It is likely that the arbitrary use of PMA and calcium ionophores may be a major confounder in the study of autoantigen citrullination in RA. (2) Neutrophils dying by LTH in the RA joint cannot be morphologically distinguished from NETosis. This may falsely suggest that NETosis is increased in RA and relevant to disease pathogenesis. (3) Citrullination in the cellular compartment of the rheumatoid joint is characterized by modification of a broad range of proteins and autoantigens (58, 165, 166) and can only be reproduced *in vitro* by membranolytic damage to the neutrophil (58). In contrast, *in vitro* studies that aimed to define a role of NETosis in autoantigen citrullination are less stringent and posit that citrullination of one or two proteins is sufficient evidence to link NETosis and RA. (4) The potential success of PAD4 deficiency and PAD inhibitors in treating experimental arthritis may be misinterpreted as *in vivo* evidence that NETosis is relevant for RA, while this effect may result from transcriptional repression of immune effector functions or inhibition of LTH.

It is the opinion of these authors that the role of NETosis in autoantigen citrullination needs to be revisited. This reappraisal needs to consider the type of stimulus used, the spectrum of citrullinated autoantigens generated during NETosis, and whether this spectrum is representative of the citrullinome in the RA joint (58, 165, 166). It further requires the presence of appropriate negative controls to confirm the specificity of citrullinated protein detection.

Depending on the mechanism that inactivates PAD4 during NETosis (reversible vs. irreversible) (71), it is possible to assume that PADs released during this process may promote citrullination of extracellular substrates (167). Nevertheless, neutrophils dying by necrosis or LTH are even more efficient than NETosis to induce citrullination of extracellular substrates (167), suggesting that NETosis itself may not be required for this process.

### Other Forms of Cell Death That May Mimic NETosis

#### Defective Mitophagy in Neutrophils

Since mitochondrial dysfunction can have deleterious effects on cells, quality control mechanisms have evolved to eliminate damaged mitochondria (168). However, while most cells use an autophagic process called mitophagy to remove damaged mitochondria, recent evidence demonstrated that mitophagy is defective in neutrophils (19). To compensate this problem, neutrophils employ two complementary pathways to achieve mitochondrial clearance. (1) Mitochondrial contents including mtDNA in complex with transcription factor A mitochondria (TFAM) are expelled from the neutrophil. This likely happens through direct fusion of the mitochondria with the plasma membrane. The release of mtDNA–protein complexes into the extracellular space is therefore a normal process in neutrophil biology. (2) If mtDNA undergoes oxidation, it dissociates from TFAM and is redirected to lysosomes for degradation (19).

Interestingly, early studies had demonstrated that expulsion of mtDNA from neutrophils can be induced by LPS or complement C5a following priming with GM-CSF (7) (Figure 4). This may indicate physiologically increased clearance of mitochondria in response to inflammatory stimuli. Due to morphological similarities with NETosis (extracellular DNA), however, these structures have been termed mitochondrial NETs. Different to actual NETs, mtDNA is released from viable neutrophils and does not appear to contain histones, neutrophil granule proteins (e.g., MPO and PR3), or antimicrobial properties (7). The analogy with NETs is therefore unsuitable and creates confusion around the biology of NETosis. Importantly, no rationale exists to posit that citrullination may have a role in the release of mtDNA–protein complexes from neutrophils, reinforcing that PAD4 is not central to any process that releases DNA and so-called NETs.

#### Neutrophil Extracellular ox-mtDNA in SLE

In 2011, three different groups suggested distinct mechanisms to explain a possible increase of NETosis in patients with SLE (83, 169, 170). While no effort was made by other groups to support or discard any of these models, two recent studies stressed that NETosis may indeed play no role in SLE pathogenesis (19, 100). Instead, the release of ox-mtDNA from neutrophils, initially mistaken for NETosis, appears to be relevant to SLE (19). While data from either study can lead to similar conclusions, some differences deserve attention.

In previous studies, freshly isolated neutrophils from patients with SLE were shown to suffer NETosis when exposed to ribonucleoprotein-containing immune complexes (RNP-ICs) *via* TLR7 and FcγRIIa ligation. This unique process was reproduced in neutrophils from healthy controls when cells were primed with type I IFN (IFN-I) to induce TLR7 expression (170). Using a similar model, Caielli and colleagues challenged this paradigm and demonstrated that defective mitophagy together with IFN priming, but not NETosis, is responsible for the release of pro-inflammatory ox-mtDNA in response to RNP-ICs (19). In this scenario, neutrophil exposure to IFN-I and RNP-ICs interferes with the disassembly of mtDNA–TFAM complexes, which are required for ox-mtDNA disposal by lysosomes. This results in ox-mtDNA accumulation in mitochondria and further expulsion as highly interferogenic ox-mtDNA–TFAM complexes (19). Different to NETosis, neutrophils remain alive while releasing ox-mtDNA in the extracellular space, gDNA is not released, and the process is dependent on mtROS, but not NADPH oxidase (19) (Figure 4). While Lood and colleagues reached similar conclusions in regard to the release of ox-mtDNA in response to RNP-ICs and the requirement of mtROS, but not NADPH oxidase, they found that IFN-I priming was not required to drive this process (100). They also described that a similar process spontaneously occurs in low-density granulocytes (LDGs), a subset of inflammatory granulocytes enriched in SLE (100).

While the studies by Caielli and Lood were performed using distinct populations of patients (pediatric vs. adult) (19, 100), it is unclear whether this may explain the difference in the requirement of type I IFN to induce TLR7, as RNP-ICs signal through this receptor (170).

Although the study by Lood and colleagues still uses the term NETosis (100), both studies are consistent with the evolving idea that this form of neutrophil death is not involved in the pathogenesis of SLE. Instead, mechanisms that enhance the production and release of ox-mtDNA may be responsible for generating critical inflammatory components that contribute to SLE pathogenesis. Importantly, these studies support the conclusions by Campbell and colleagues in lupus-prone mice deficient in Nox2 (the p91^phox^ component of the phagocyte NADPH oxidase), which demonstrated that NETosis does not contribute to lupus *in vivo*. Since Nox2 deficiency exacerbates lupus in these mice, NETosis, or some other activity linked to Nox2, instead acts to inhibit disease pathogenesis (99). This fascinating idea is further supported by the observation that deficiencies in NADPH oxidase activity in humans are associated with SLE, lupus-like disease, lupus autoantibodies, and a systemic IFN-I signature (100, 171–175). Neutrophils from these patients are resistant to NETosis, but have fully conserved mtROS activity (176), which together may predispose to SLE. Finally, individuals with NADPH oxidase deficiency have an increased risk of fungal and bacterial infections (176), underscoring that the sole expulsion of mtDNA or ox-mtDNA is not antimicrobial and should not be considered a form of NETosis. As NADPH oxidase appears to be protective for the development of SLE, it is possible that this enzyme complex regulates the production or clearance of neutrophil ox-mtDNA. Indeed, strong evidence supports an interplay between mitochondria and NADPH oxidase that may provide both feed-forward and feedback regulations in the production of ROS (177). Moreover, it is interesting to highlight that the extracellular release of mtDNA induced by GM-CSF and LPS/C5a is dependent on NADPH oxidase (7), supporting that this enzyme complex has a role in the maintenance of mitochondria in neutrophils. NETosis could moreover be protective for SLE by degrading cytokines and chemokines (178). The finding that the inhibition of NETosis by PTU induces the production of anti-MPO antibodies and vasculitis in rats (98) further supports a protective role of NETosis in autoimmunity. Defining the role of NADPH oxidase in SLE has therefore implications for both disease pathogenesis and the development of therapeutic strategies.

## Concluding Remarks

The biochemical and functional distinction of NETosis, LTH, and defective mitophagy is critical to understanding the distinct role of these processes for human immunity and autoimmunity. While the extracellular release of neutrophil DNA induced by various stimuli may appear morphologically similar, they seem to be consequences of opposing evolutionary forces. Here, we propose the existence of at least three distinct mechanisms that drive the active extrusion of neutrophil DNA (Figure 4), which are as follows: (1) NETosis, which represents a hardwired cellular program that likely evolved to kill pathogens, (2) LTH, which is the cellular consequence of successful strategies that evolved in specific pathogens to kill neutrophils (membranolysis), and (3) the expulsion of mtDNA, which represents a normal function in neutrophil biology to clear the cell of damaged mitochondrial material, but can generate ox-mtDNA in the SLE environment. While these processes can be easily studied by defining the effects of PMA, calcium ionophores and IFN-I plus RNP-ICs on neutrophils, it is important to emphasize that these stimuli are artificial. They are most likely non-representative of the complexity of signals that converge in the neutrophil during infection or autoimmunity. We anticipate that variations of these mechanisms exist *in vivo*.

The attempt to derive a biochemical definition of the distinct processes that release DNA from neutrophils offers a unique opportunity to reevaluate the role of PAD4 and NETosis as drivers and potential targets in human diseases. Based on this model, the use of PAD inhibitors may be indicated to block the detrimental effects of LTH, but not NETosis. First, PAD inhibitors could act as “antibiotics” to prevent ongoing hypercitrullination induced by bacterial toxins, thus allowing bactericidal NETosis to occur. The finding that the PAD inhibitor Cl-amidine improves survival in a murine model of sepsis strongly supports this hypothesis (151). Second, they may decrease the burden of citrullinated autoantigens in RA without affecting NETosis, which has been shown to have distinct anti-inflammatory activities (178). Given that ionomycin and perforin induce hyperactivation of PAD2 and PAD4 in neutrophils, it is conceivable that blocking LTH may require inhibition of both enzymes. Third, PAD4 inhibitors may have an indication to treat autoimmune diseases as transcriptional repressors of immune effector functions, but not under the rationale that these molecules act by blocking NETosis. In SLE, the use of PAD4 inhibitors to block the production and release of ox-mtDNA has no rationale, unless it is demonstrated that this enzyme is essential for this process. Blocking the production of mtROS, however, may be reasonable.

Besides infectious and autoimmune diseases (e.g., SLE) that have been associated with decreased production of NETosis (as consequence of NADPH oxidase deficiency) (100, 171–176), diseases related to an increased neutrophil propensity to undergo NETosis need to be carefully defined. A better biochemical definition of the diverse mechanisms that can affect neutrophil function should allow for the identification of novel biomarkers and help with the classification of disease entities that may benefit from targeting those pathways. There remains a need to clarify the true role of NETosis in human health and disease.

## Ethics Statement

Informed consent was obtained from all individuals as approved by the Johns Hopkins Institutional Review Board.

## Author Contributions

All authors listed have made substantial, direct, and intellectual contribution to the work and approved it for publication.

## Conflict of Interest Statement

FA received a grant from Medimmune. MK declares no conflict of interest.
